# Lipopolysaccharide-Induced Spatial Memory and Synaptic Plasticity Impairment Is Preventable by Captopril

**DOI:** 10.1155/2016/7676512

**Published:** 2016-10-18

**Authors:** Azam Abareshi, Akbar Anaeigoudari, Fatemeh Norouzi, Mohammad Naser Shafei, Mohammad Hossein Boskabady, Majid Khazaei, Mahmoud Hosseini

**Affiliations:** ^1^Neurocognitive Research Center, Faculty of Medicine, Mashhad University of Medical Sciences, Mashhad, Iran; ^2^Department of Physiology, School of Medicine, Jiroft University of Medical Sciences, Jiroft, Iran; ^3^Department of Physiology, Esfarayen Faculty of Medical Sciences, Esfarayen, Iran; ^4^Neurogenic Inflammation Research Center, Faculty of Medicine, Mashhad University of Medical Sciences, Mashhad, Iran

## Abstract

*Introduction.* Renin-angiotensin system has a role in inflammation and also is involved in many brain functions such as learning, memory, and emotion. Neuroimmune factors have been proposed as the contributors to the pathogenesis of memory impairments. In the present study, the effect of captopril on spatial memory and synaptic plasticity impairments induced by lipopolysaccharide (LPS) was investigated.* Methods.* The rats were divided and treated into control (saline), LPS (1 mg/kg), LPS-captopril (LPS-Capto; 50 mg/kg captopril before LPS), and captopril groups (50 mg/kg) before saline. Morris water maze was done. Long-term potentiation (LTP) from CA1 area of hippocampus was assessed by 100 Hz stimulation in the ipsilateral Schaffer collateral pathway.* Results.* In the LPS group, the spent time and traveled path to reach the platform were longer than those in the control, while, in the LPS-Capto group, they were shorter than those in the LPS group. Moreover, the slope and amplitude of field excitatory postsynaptic potential (fEPSP) decreased in the LPS group, as compared to the control group, whereas, in the LPS-Capto group, they increased compared to the LPS group.* Conclusion.* The results of the present study showed that captopril improved the LPS-induced memory and LTP impairments induced by LPS in rats. Further investigations are required in order to better understand the exact responsible mechanism(s).

## 1. Introduction

Renin-angiotensin system (RAS) is one of the neuropeptide systems in the brain. The substrate of RAS, angiotensinogen, is cleaved by the renin enzyme to form the decapeptide angiotensin (Ang I) in the brain [1]. Ang I is then converted to an octapeptide, Ang II, by angiotensin converting enzyme (ACE) [2] which is extensively located within various areas of central nervous system (CNS) [3]. Ang II is cleaved by glutamyl aminopeptidase A (AP-A) to form heptapeptide, Ang III. Ang II can also be cleaved to Ang (1-7) by carboxypeptidase P [2]. In addition, ACE2 acts on Ang I and Ang II to form Ang 1-9 and Ang 1-7, respectively. ACE2 has been shown to have a higher efficiency for conversion of Ang II to Ang 1-7 than for conversion of Ang I to Ang 1-9. This enzyme has been expressed in a low concentration in the CNS [4]. The main effector of RAS, Ang II, binds to specific receptors in the brain to induce multiple actions [5]. It also regulates blood pressure, sodium and water balance, and sexual behaviors [2, 6]. The brain RAS has been shown to be involved in memory loss associated diseases such as Alzheimer's disease (AD) [1, 7] and cognitive dysfunctions which are preventable by angiotensin converting enzymes (ACE) inhibitors including captopril [1, 8]. Long-term potentiation (LTP), one of the major forms of activity dependent synaptic plasticity, is the primary experimental model for evaluating the synaptic basis of learning and memory in the hippocampus of vertebrates [9, 10]. An enhanced level of Ang II has been reported to be able to inhibit LTP induction in hippocampus [8].

In addition, RAS has been proposed to have a role in inflammatory responses and lipopolysaccharide- (LPS-) mediated microglial activation [11]. On the other hand, ACE inhibitors such as captopril have also been reported to have anti-inflammatory effects both in vitro and in vivo through reducing inflammatory cytokines such as tumor necrosis factor *α* (TNF*α*) and interleukin 1 (IL-1) [12, 13].

LPS, a potent inflammation-inducing agent in experimental studies, mimics the role of live bacteria and affects cognition and induces sickness behaviors when administered systemically or centrally [14]. These effects are attributed to overproduction of cytokines including interleukin-1*β* (IL-1*β*) and TNF*α* from immune cells [15]. Additionally, brain tissues oxidative damage has been reported to have an important role in learning and memory impairments induced by LPS [16]. Interestingly, an increased level of malondialdehyde (MDA) as an index of oxidative stress and a reduced level of total thiol content had been accompanied with increase in IL-1*β*, cognitive dysfunction, spatial learning deficits in Morris water maze (MWM), and synaptic plasticity impairment followed by LPS administration [14, 16]. It has also been reported that captopril is able to increase blood brain barrier permeability in rats [17]. Captopril also affects generation of proinflammatory and anti-inflammatory cytokines induced by LPS [18, 19]. We, therefore, decided to test whether captopril can prevent LPS-induced spatial memory and synaptic plasticity impairments.

## 2. Materials and Methods

### 2.1. Animals and Drugs

**Figure 1:**
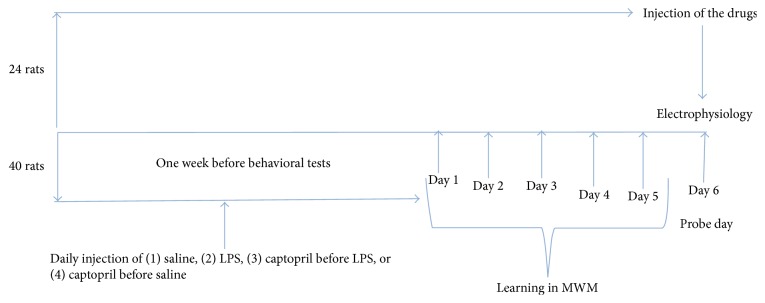
The protocol for the experiments.

Male Wistar rats, 12 weeks old (240 ± 10 g), were purchased from the animal house of Mashhad University of Medical Sciences, Mashhad, Iran. The animals were housed in standard conditions (temperature 22 ± 2°C and 12 h light/dark cycle). The rats had free access to food and water. The animals were treated in accordance with approved procedures by the Committee on Animal Research of Mashhad University of Medical Sciences. Forty of the animals were divided into four groups (*n* = 10 in each group) and used for behavioral studies: (1) control, (2) LPS, (3) LPS-captopril (LPS-Capto), and (4) Capto groups. The animals in the LPS and LPS-Capto groups were treated by LPS (1 mg/kg; i.p.) [20], which began one week prior to the behavioral tests and continued to be injected 2 h before each trial of MWM test (Figure 1). The animals in the control and Capto groups received 1 mL/kg of saline instead of LPS. In the LPS-Capto and Capto groups, 50 mg/kg of captopril (i.p.) [21–23] was daily injected one week prior to start of the experiments and also was injected 30 min before LPS or saline. It has also been reported that captopril is bale to increase blood brain barrier permeability in rats [17]. The rest of the animals [23] were grouped into (1) control, (2) LPS, and (3) LPS-Capto (*n* = 8 in each group) and used for electrophysiological experiments after receiving a single dose of drugs or vehicle. LPS was purchased from Sigma (Sigma Chemical Co.). Captopril was provided by Daroupakhsh Company, Iran.

### 2.2. Morris Water Maze (MWM) Test

MWM apparatus was made of a circular black pool (136 cm diameter, 60 cm high, and 30 cm deep) with boundaries of the four quadrants including Q1 (northwest), Q2 (northeast), Q3 (southwest), and Q4 (southeast) that was filled with water (23–25°C). A circular platform (10 cm diameter and 28 cm high) was hidden within the pool approximately 2 cm below the surface of the water in the center of the northwest quadrant. To determine the path, visual cues were fixed at several locations around the room outside the maze. The path, time, and speed of the animals to find the platform were traced by a camera. Before each experiment, the rats were familiarized with the water maze without a platform for 30 seconds. The animals performed four trials each day for five consecutive days and, in each trial, they were released randomly at one of the four positions. In each trial, the rat was allowed to swim until it found and remained on the platform for 20 seconds. If the animal was not able to find the platform within 60 seconds, it was guided to the platform by the experimenter and allowed to stay on it for 20 seconds. After removing from the pool, it was dried and placed in the cage for another 20 seconds. The time spent and distance traveled to reach the platform were recorded by a video tracking system. On the sixth day, the platform was removed, and the animals were allowed to swim for 60 seconds. The time spent and path traveled in the target quadrant (Q1) were compared between the groups.

### 2.3. Electrophysiological Study

For electrophysiological experiments, 24 of the animals were divided into three groups: (1) control, (2) LPS, and LPS-Capto (*n* = 8 in each group). The animals were anesthetized with urethane (1.6 g/kg) and their heads were then fixed in a stereotaxic apparatus. After exposing the skull, two small holes were drilled, under sterile conditions, to place stimulating and recording electrodes. Field potential was recorded from CA1 area of hippocampus. For this purpose, a bipolar stimulating electrode (stainless steel, 0.125 mm diameter, AM system) was infixed in the ipsilateral Schaffer collateral pathway (AP = 3 mm; ML = 3.5 mm; DV = 2.8–3 mm) and a unipolar recording electrode was lowered into the stratum radiatum of right CA1 area of hippocampus (AP = 4.1 mm; ML = 3 mm; DV = 2.5 mm). To ensure proper placement of the electrodes, physiological and stereotaxic indicators were used. Paired pulse facilitation (PPF) was considered as physiological indicator, and coordinates obtained from atlas of Paxinos and Watson were considered as stereotaxic indicators. PPF was measured by delivering ten consecutive evoked responses of paired pulses at 50 ms interpulse interval to the Schaffer collateral pathway at frequency 0.1 Hz (10 s interval). The stimulating electrode was connected to a stimulator and recording electrode was connected to an amplifier. Obtained extracellular field potential from CA1 area of hippocampus following stimulation of the Schaffer collateral pathway was amplified (100x) and filtered (1 Hz to 3 kHz band pass) using differential amplifier. A maximum field excitatory postsynaptic potential (fEPSP) was obtained by stimulating the Schaffer collateral pathway and recording in CA1 area. After a 30 min stabilization period, in order to evaluate synaptic potency before induction of LTP, an input-output (I/O) function was exerted by gradually increasing the stimulus intensities with constant current (input) and recording fEPSP (output). A baseline recording was then taken at 30 min before induction of LTP. After ensuring a steady state baseline response, in order for LTP induction, a high frequency stimulus (HFS) protocol of 100 Hz was applied. The stimuli with the intensities which produced 50% of the maximum response were applied to induce LTP.

The fEPSP was then recorded for 90 min after high frequency stimuli. Computer-based stimulation and recording were performed using Neurotrace software version 9 and Eletromodule 12 (Science Beam Institute, Tehran, Iran), respectively. The values of the slope and amplitude of the fEPSP were averaged of the 10 consecutive traces. Reponses were analyzed using custom software from the same institute.

### 2.4. Statistical Analysis

All data were expressed as means ± SEM and analyzed using two-way ANOVA followed by Tukey's post hoc test. Differences were considered statistically significant when *P* < 0.05.

## 3. Results

### 3.1. MWM Results

**Figure 2:**
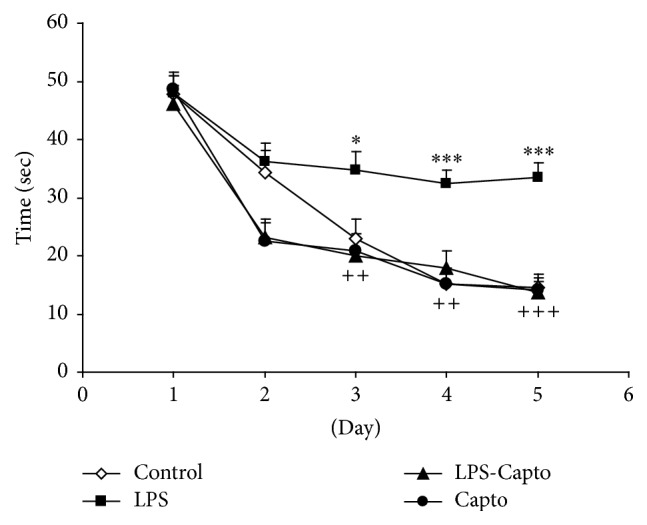
Comparison of time latency to reach the platform in the Morris water maze test between the four groups. Data are presented as mean ± SEM (*n* = 10 in each group). ^*∗*^ *P* < 0.05 and ^*∗∗∗*^ *P* < 0.001 compared with the control group and ^++^ *P* < 0.01 and ^+++^ *P* < 0.001 compared with the LPS group.

Using two-way ANOVA, the results showed that the treatment significantly affected the escape latency to reach the platform (*f*_(3,767)_ = 23.28; *P* < 0.001). There were also significant effects for days on the escape latency to reach the platform (*f*_(4,767)_ = 64.04; *P* < 0.001). There was a significant interaction between the treatment and days on the escape latency to reach the platform (*f*_(12,767)_ = 2.50; *P* < 0.01). The results also showed that the escape latency to reach the platform in the LPS group was significantly higher than that in the control group at days 3 (*P* < 0.05), 4 (*P* < 0.001), and 5 (*P* < 0.001). The animals in the LPS-Capto group had a significantly shorter time latency to reach the platform in comparison to those of the LPS group at days 3 (*P* < 0.01), 4 (*P* < 0.01), and 5 (*P* < 0.001). There was no significant difference in the time spent to reach the platform between the control and LPS-Capto groups. There was also no significant difference between the Capto and control groups (Figure 2).

**Figure 3:**
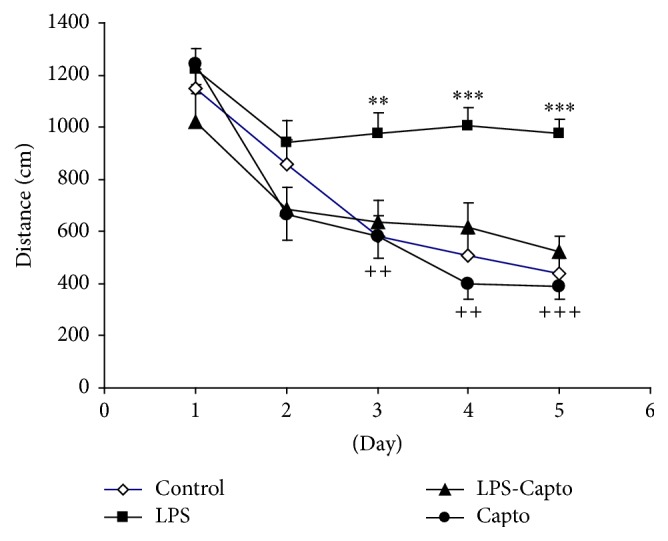
Comparison of the distance traveled to reach the platform in the Morris water maze test between the four groups. Data are presented as mean ± SEM (*n* = 10 in each group). ^*∗∗*^ *P* < 0.01 and ^*∗∗∗*^ *P* < 0.001 compared with the control group and ^++^ *P* < 0.01 and ^+++^ *P* < 0.001 compared with the LPS group.

Using two-way ANOVA, the results showed that the treatment significantly affected the distance traveled to reach the platform (*f*_(3,767)_ = 23.56; *P* < 0.001). There were also significant effects for days on the distance traveled to reach the platform (*f*_(4,767)_ = 34.20; *P* < 0.001). There was a significant interaction between the treatment and days on the distance traveled to reach the platform (*f*_(12,767)_ = 2.45; *P* < 0.01). The results also showed that the distance traveled to reach the platform in the LPS group was significantly higher than that in the control group at days 3 (*P* < 0.01), 4 (*P* < 0.01), and 5 (*P* < 0.001). The animals had a significantly shorter traveled distance to reach the platform in the LPS-Capto group in comparison to the LPS group at days 3 (*P* < 0.01), 4 (*P* < 0.01), and 5 (*P* < 0.001). There was no significant difference in the length of the swimming path between the control and LPS-Capto groups. There was also no significant difference between the Capto and control groups (Figure 3).

**Figure 4:**
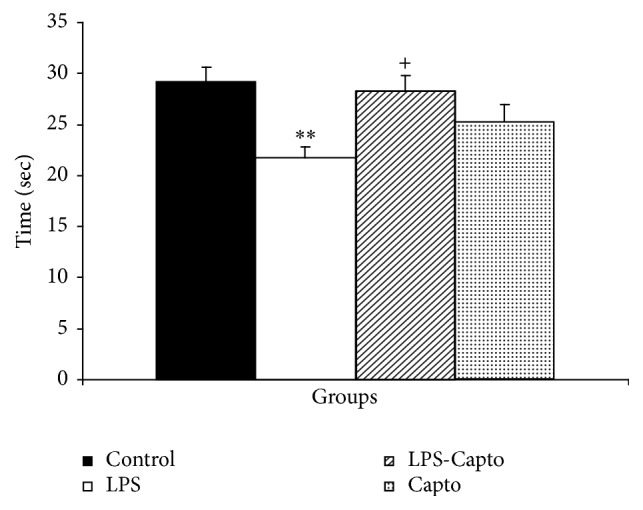
The results of the time spent in the target quadrant (Q1) in probe day, 24 hours after the last learning session. The platform was removed and the time spent in the target quadrant was compared between the groups. Data are shown as mean ± SEM (*n* = 10 in each group). ^*∗∗*^ *P* < 0.01 compared with the control group and ^+^ *P* < 0.01 compared with the LPS group.

**Figure 5:**
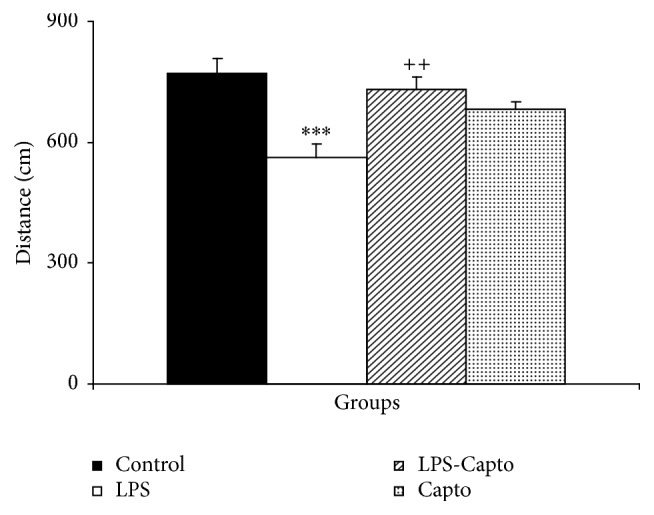
The results of the distance traveled in the target quadrant (Q1) in probe day, 24 hours after the last learning session. The platform was removed and the distance traveled in the target quadrant was compared between the groups. Data are shown as mean ± SEM (*n* = 10 in each group). ^*∗∗∗*^ *P* < 0.001 compared with the control group and ^++^ *P* < 0.01 compared with the LPS group.

In the probe day, the animals of the LPS group spent lower time (*P* < 0.001) and traveled shorter distance (*P* < 0.001) in the target quadrant (Q1) than those of the control group. The animals in the LPS-Capto group spent greater time and traveled longer distance in the Q1 compared to those in the LPS group (*P* < 0.05 and *P* < 0.01, resp.). There was no significant difference in the time spent and distance traveled in the Q1 between the control and LPS-Capto groups. The results also showed that there was no significant difference in the time spent and distance traveled in the target quadrant between the Capto and control groups (Figures 4 and 5).

### 3.2. Electrophysiological Results

**Figure 6:**
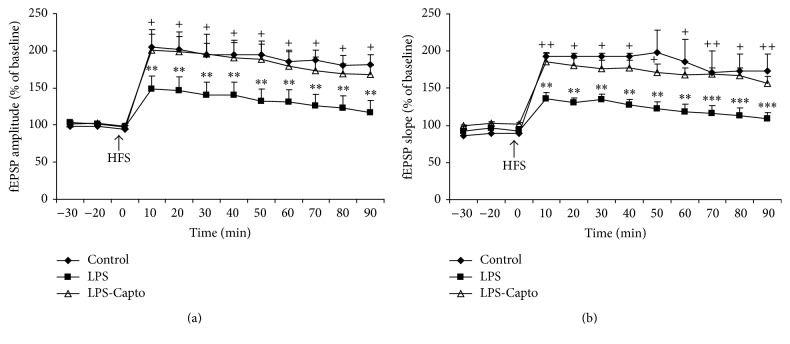
The results of LTP induction in CA1 area of the hippocampus using 100 Hz tetanic stimulation at (a) the fEPSP amplitude and (b) the fEPSP slope. Data are presented as the average percentage changes from baseline responses. Each point shows mean ± SEM (*n* = 8 in each group). The amplitude and slope of fEPSP in the LPS group were lower than those in the control group (^*∗∗*^ *P* < 0.01 and ^*∗∗∗*^ *P* < 0.001) and in the LPS-Capto group they were higher with respect to the LPS group (^+^ *P* < 0.05 and ^++^ *P* < 0.01).

After inducing HFS, the mean fEPSP amplitude in the LPS group decreased significantly with respect to the control group (*P* < 0.01). The mean fEPSP amplitude in the LPS-Capto group was significantly higher than that in the LPS group (*P* < 0.05). There was no significant difference in fEPSP amplitude between the control and LPS-Capto groups (Figure 6(a)). In addition, after applying HFS, the fEPSP slope in the LPS group was significantly lower than that in the control group (*P* < 0.01). Injection of captopril increased the mean fEPSP slope in the LPS-Capto group in comparison to the LPS group (*P* < 0.05); however, there was no significant difference in fEPSP slope between the control and LPS-Capto groups (Figure 6(b)).

## 4. Discussion

Previous studies have demonstrated that LPS impairs learning and memory [14, 24]. In parallel with such reports, in the current study, intraperitoneal injection of LPS also impaired spatial learning and memory in the Morris water maze [25]. The results showed that the animals of the LPS group had more time latency (Figure 2) and longer traveled distance (Figure 3) to find the escape platform compared with those of the control group. The results of probe trial also showed that the animals of the LPS group did not well look for the location of the escape platform and spent less time (Figure 4) and traveled shorter distance (Figure 5) in the target quadrant (Q1) with respect to those of the control group.

LTP is a form of activity dependent synaptic plasticity which is suggested to be a predominant mechanism of learning and memory processes [9]. In hippocampus, LTP induction has been well known as a principle experimental model for studying synaptic basis of learning and memory in vertebrates [26]. In previous studies, LPS administration has resulted in suppression of LTP induction in rat dentate gyrus in vitro [27] and subiculum in vivo [28]. In the current study, LPS administration also impaired LTP induction in rats' hippocampus which was reflected by decreasing of amplitude (Figure 6(a)) and slope (Figure 6(b)) of fEPSP in the LPS group compared to the control group.

Deleterious effects of LPS on neuronal function such as synaptic plasticity, learning, and memory have been attributed to inflammatory responses and overproduction of proinflammatory cytokines including TNF*α* and IL-1*β* [29]. Experimental findings have indicated that serum level of TNF*α*, IL-1*β*, and IL-6 increases after LPS administration [14]. In addition, it has been reported that detrimental effects of LPS, IL-1*β*, and IL-6 on spatial learning and memory are probably mediated by inhibiting LTP induction in the hippocampus [14, 30]. Considering these facts, it seems that an excessive production of proinflammatory cytokines followed by injection of LPS plays an important role in spatial memory and synaptic plasticity deficits caused by LPS in the present study. Supporting this idea, we have previously shown that administration of LPS (1 mg/kg) increases serum TNF*α* levels [10, 16, 31].

In addition, the results of our study also indicated that intraperitoneal administration of captopril 30 min before LPS diminished harmful effects of LPS on spatial learning and memory and synaptic plasticity. In the current study, behavioral results revealed that the animals of the LPS-Capto group not only had a lower latency (Figure 2) and shorter traveled distance (Figure 3) to find the escape platform in comparison with those of the LPS group but also spent more time (Figure 4) and traveled longer distance (Figure 5) to look for the location of the platform in the target quadrant in probe day. In electrophysiological experiments, administration of captopril also enhanced both the amplitude (Figure 6(a)) and the slope (Figure 6(b)) of fEPSP.

RAS system is one of the neuropeptide systems in the brain that is considered to have some effects on neuronal functions [32]. RAS of the brain has been proposed to be involved in processing of sensory information, learning and memory, and regulation of emotional behaviors [7, 33]. Researches have suggested that an increased level of RAS activity is accompanied with cognitive functions impairments. It has also been reported that injection of Ang II or renin into the CNS disturbs retention of passive avoidance tasks [34]. In addition, Ang II and its specific analogues inhibited LTP induction and spatial learning when administered into the hippocampus [4]. On the other hand, ACE inhibitors such as captopril and perindopril were able to increase conditioned avoidance and habituation memory [1]. It has been demonstrated that intraperitoneal and intracerebroventricular injection of captopril improved cognitive processes in radial 8 arm maze and Y maze paradigms [35]. Sepehri et al. also confirmed that captopril improved spatial memory of aged rats [36]. Captopril was also reported to be able to block trimethyltin-induced spatial memory deficits in rats [37]. According to these facts, an increased level of RAS activity followed by LPS injection which was restored by captopril might be suggested in development of the results of the current study. However, more researches are needed to be done to elucidate this subject. Ability of captopril to pass from brain barrier may elucidate central acting effects of the drug which was seen in the present study [38, 39].

In recent studies, activation of RAS has been exhibited to have a significant proinflammatory action. It has been indicated that locally produced Ang II by inflamed vessels promotes synthesis and secretion of inflammatory cytokines such as IL-6 [40]. The results of previous studies have confirmed that administration of LPS increases RAS activity which is reflected by an enhanced level of Ang II in the plasma of rats [41]. On the other hand, anti-inflammatory effects of certain ACE inhibitors have been reported in both in vivo and in vitro studies [42]. Captopril, as a well-known ACE inhibitor, has been demonstrated to inhibit LPS-induced inflammatory responses [18]. It has also been reported that pretreatment with captopril suppresses expression of inflammatory cytokines such as TNF*α* in rabbits [43]. Captopril has also been shown to increase concentration of anti-inflammatory cytokines such as IL-10 [37]. Considering this scientific evidence, improving effects of captopril on spatial memory and synaptic plasticity observed in the present study may be, at least in part, due to inhibition of production of proinflammatory cytokines, which needs, however, to be more evaluated.

Additionally, the reactive oxygen species (ROS) and brain tissues oxidative damage play an important role in learning and memory impairment [44]. The RAS is also proposed to have a crucial implication in induction of ROS [45]. Previous studies indicated that produced Ang II by vascular tissues enhances the production of ROS via activating AT_1_ receptors [46]. It has been reported that chronic activation of the brain RAS with sustained generation of Ang II causes cardiovascular remodeling, inflammation responses, and oxidative stress leading to endothelial dysfunction and, finally, disrupts regulation of cerebral blood flow [4]. It has also been documented that age-related cognition deficits are associated with the stimulation of AT_1_, reduction of cerebral blood flow, and enhancement of oxidative stress [47]. Previously, we also suggested a role for the brain tissues oxidative damage in memory impairment following peripheral LPS administration [16]. On the other hand, treatment with ACE inhibitors such as captopril has been proposed to enhance the activities of antioxidant enzymes as well as nonenzymatic antioxidant defense [48, 49]. Captopril is able to scavenge free radicals and also is able to inhibit reactive oxygen and nitrogen species production [50]. It has also been reported that captopril pulls up GSH depletion and GSSG formation caused by doxorubicin [51]. Given these facts, it seems that oxidative stress following administration of LPS along with excessive activation of RAS accounted for the development of the results of the present study. It also seems that inhibition of LPS-induced spatial memory and synaptic plasticity impairments by captopril is in part by preventing the brain tissues oxidative damage. However, these mechanisms should be clarified in the future.

In summary, it seems that administration of LPS enhances RAS activity which impairs spatial memory and synaptic plasticity. The results of the present study showed that pretreatment with captopril prevented LPS-induced spatial learning and memory and synaptic plasticity impairments, confirming a relationship between RAS and LPS-induced brain dysfunctions.
